# Correction: The corrosion behaviors of multilayer diamond-like carbon coatings: influence of deposition periods and corrosive medium

**DOI:** 10.1039/d1ra90166d

**Published:** 2021-11-19

**Authors:** Mingjun Cui, Jibin Pu, Guangan Zhang, Liping Wang, Qunji Xue

**Affiliations:** State Key Laboratory of Solid Lubrication, Lanzhou Institute of Chemical Physics, Chinese Academy of Sciences Lanzhou 730000 China lpwang@licp.cas.cn qjxue@lzb.ac.cn +86 931 4968163 +86 931 4968080; Key Laboratory of Marine Materials and Related Technologies, Ningbo Institute of Materials Technology and Engineering, Chinese Academy of Sciences Ningbo 315201 China; University of Chinese Academy of Sciences Beijing 100039 China

## Abstract

Correction for ‘The corrosion behaviors of multilayer diamond-like carbon coatings: influence of deposition periods and corrosive medium’ by Mingjun Cui *et al.*, *RSC Adv.*, 2016, **6**, 28570–28578, DOI: 10.1039/C6RA05527C.

The authors regret that an incorrect version of Fig. 3 was included in the original article. The correct version of Fig. 3 is shown below.

**Fig. 3:**
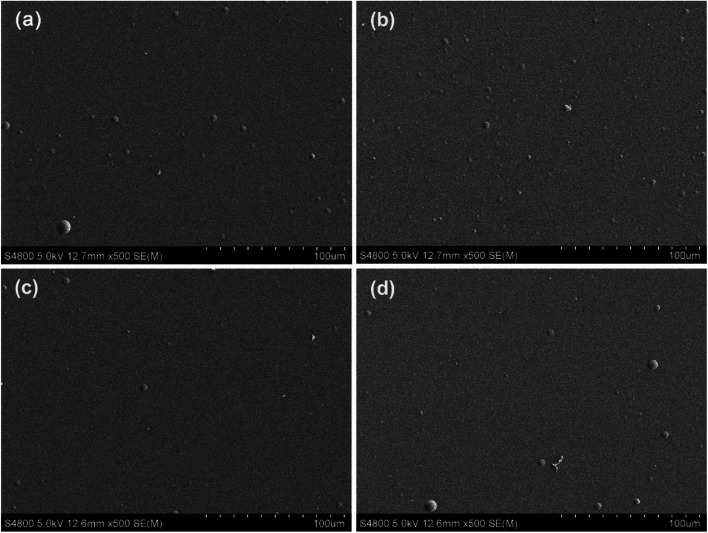
SEM images of surface morphology of multilayer DLC coatings. (a) 5 deposition periods, (b) 12 deposition periods, (c) 15 deposition periods, (d) 20 deposition periods.

The Royal Society of Chemistry apologises for these errors and any consequent inconvenience to authors and readers.

## Supplementary Material

